# A statistical framework for system-level synthesis

**DOI:** 10.64898/2026.10.19.760244

**Published:** 2026-10-21

**Authors:** M. Senthil Kumar, Filip Garbicz, Rubén Carrasco, Rafael A. Irizarry

## Abstract

Simultaneous profiling of thousands of biological features is now routine. Yet transforming the data into interpretable models of system behavior remains a fundamental bottleneck as current analyses generally identify feature-wise associations or extract latent structure without anchoring it to the variables that define the scientific question. Here we propose a framework for system-level synthesis that learns and predicts overarching processes, and reliably identifies participating features without data reuse. We develop Smoothing Spline Analysis of Variance–Singular Value Decomposition to learn overarching processes as smooth, flexible functions of nominal, ordinal and continuous covariates and their interactions. This unlocks prediction from covariates alone, while reserving measurements for conditional inference of feature participation. In applications, the framework recovered established biology while generating predictions and testable hypotheses. By linking high-dimensional variation to covariates, the framework advances analysis beyond feature-wise associations and unsupervised patterns, toward interpretable system-level models that vary with biological and experimental context.

## Full Text Availability

The license terms selected by the author(s) for this preprint version do not permit archiving in PMC. The full text is available from the preprint server.

